# Serum levels of the chemokine CCL2 are elevated in malignant pleural mesothelioma patients

**DOI:** 10.1186/s12885-019-6419-1

**Published:** 2019-12-10

**Authors:** Takumi Kishimoto, Nobukazu Fujimoto, Takeshi Ebara, Toyonori Omori, Tetsuya Oguri, Akio Niimi, Takako Yokoyama, Munehiro Kato, Ikuji Usami, Masayuki Nishio, Kosho Yoshikawa, Takeshi Tokuyama, Mouka Tamura, Yoshifumi Yokoyama, Ken Tsuboi, Yoichi Matsuo, Jiegou Xu, Satoru Takahashi, Mohamed Abdelgied, William T. Alexander, David B. Alexander, Hiroyuki Tsuda

**Affiliations:** 1 Japan Organization of Occupational Health and Safety, Research Center for Asbestos-related Diseases, Okayama Rosai Hospital, Okayama, Japan; 2 Department of Occupational and Environmental Health, Nagoya City University Graduate School of Medical Sciences, Nagoya, Japan; 3 Department of Healthcare Policy and Management, Nagoya City University Graduate School of Medical Sciences, Nagoya, Japan; 4 Department of Respiratory Medicine, Allergy and Clinical Immunology, Nagoya City University Graduate School of Medical Sciences, Nagoya, Japan; 5 Japan Organization of Occupational Health and Safety, Department of Respiratory Medicine, Asahi Rosai Hospital, Owariasahi, Japan; 6 Department of Respiratory Medicine, Daido Hospital, Nagoya, Japan; 7 Department of Internal Medicine, Saiseikai Chuwa Hospital, Sakurai, Nara, Japan; 8 Department of Internal Medicine, National Hospital Organization Nara Medical Center, Nara, Japan; 9 Department of Medicine and Physical Medicine and Rehabilitation, Nagoya City Koseiin Medical Welfare Center, Nagoya, Japan; 10 Department of Gastroenterological Surgery, Nagoya City University Graduate School of Medical Sciences, Nagoya, Japan; 11 Department of Immunology, College of Basic Medical Sciences, Anhui Medical University, Hefei, China; 12 Nanotoxicology Project Lab, Nagoya City University, 3-1 Tanabedohri, Mizuho-ku, Nagoya, 467-8603 Japan; 13 Department of Experimental Pathology and Tumor Biology, Nagoya City University Graduate School of Medical Sciences, Nagoya, Japan; 14 Department of Forensic Medicine and Toxicology, Faculty of Veterinary Medicine, Beni-Suef University, Beni-Suef, Egypt

**Keywords:** Asbestos, Cancer, Malignant pleural mesothelioma, CCL2

## Abstract

**Background:**

Malignant pleural mesothelioma (MPM) is a debilitating disease of the pleural cavity. It is primarily associated with previous inhalation of asbestos fibers. These fibers initiate an oxidant coupled inflammatory response. Repeated exposure to asbestos fibers results in a prolonged inflammatory response and cycles of tissue damage and repair. The inflammation-associated cycles of tissue damage and repair are intimately involved in the development of asbestos-associated cancers. Macrophages are a key component of asbestos-associated inflammation and play essential roles in the etiology of a variety of cancers. Macrophages are also a source of C-C motif chemokine ligand 2 (CCL2), and a variety of tumor-types express CCL2. High levels of CCL2 are present in the pleural effusions of mesothelioma patients, however, CCL2 has not been examined in the serum of mesothelioma patients.

**Methods:**

The present study was carried out with 50 MPM patients and 356 subjects who were possibly exposed to asbestos but did not have disease symptoms and 41 healthy volunteers without a history of exposure to asbestos. The levels of CCL2 in the serum of the study participants was determined using ELISA.

**Results:**

Levels of CCL2 were significantly elevated in the serum of patients with advanced MPM.

**Conclusions:**

Our findings are consistent with the premise that the CCL2/CCR2 axis and myeloid-derived cells play an important role in MPM and disease progression. Therapies are being developed that target CCL2/CCR2 and tumor resident myeloid cells, and clinical trials are being pursued that use these therapies as part of the treatment regimen. The results of trials with patients with a similar serum CCL2 pattern as MPM patients will have important implications for the treatment of MPM.

## Background

A causal association between exposure to at least some types of asbestos and lung carcinomas and malignant pleural mesothelioma (MPM) has been long recognized [1], and in 2012 the WHO/International Agency for Research on Cancer (IARC, Lyon) classified all forms of asbestos (chrysotile, crocidolite, amosite, tremolite, actinolite, and anthophyllite) as carcinogenic to humans [2]. The 2014 updated Helsinki Criteria notes that while the use of asbestos is banned in many industrialized countries, the global production of asbestos remains at over two million metric tons a year, with an estimated 125 million people being exposed to asbestos in the workplace [3]. Furthermore, workers engaged in cleaning debris at sites of natural disasters and workers involved in demolition work may be exposed to asbestos. For example, asbestos-related disease is predicted to be significant in workers engaged in debris cleaning operations after the Great Hanshin Earthquake that occurred in Japan in 1995. Worldwide, asbestos exposure results in an estimated 255,000 deaths annually, with a significant fraction (over 30,000 in 2016) of these deaths due to mesothelioma [4]. In Japan, the number of patients that die of MPM is currently 1500 a year (Vital Statistics, Ministry of Health Labour and Welfare, Japan, 2015), and the incidence of MPM is predicted to remain relatively high in the coming years due to past exposure to asbestos.

Macrophages are considered to be essential constituents of many types of solid tumors [5, 6], and mesotheliomas are heavily infiltrated by macrophages [7–10]. The subtypes of macrophages within a tumor is heterogeneous [11]; in general however, tumor development is associated with the presence of macrophages with M2-like characteristics, particularly in patients with a poor prognosis [8, 12–14]. M2-like macrophages function in the resolution of inflammation and in protection and repair of damaged tissue [15–18]. One of the basic functions of M2-like macrophages that is associated with tissue protection and repair is immunosuppression [11], and tumors have generally been found to contain macrophages with immunosuppressive characteristics [5, 19–22].

Another important myeloid cell population that is associated with tumors are myeloid-derived suppressor cells, and there is almost universal agreement that accumulation of myeloid cells with MDSC-like phenotypes in the blood or tumor correlates with disease progression, poor prognosis, poor response to therapy, and decreased overall survival [23–29]. MDSCs are associated with tumor progression in mouse models of mesothelioma [30–32], and MDSCs are believed to be associated with mesotheliomas in human patients [33, 34].

C-C motif chemokine ligand 2 (CCL2), also known as monocyte chemotactic protein-1 (MCP-1), is expressed in most human cancers [35–37], and plays a key role in the recruitment of macrophages and MDSCs [35, 36, 38–40]. In general agreement with the findings that tumors accumulate macrophages and MDSCs that have pro-tumorigenic properties and express CCL2 and that CCL2 expression in tumor tissue is associated with advanced tumor stage and worse prognosis, there are several studies that report elevated levels of CCL2 in the serum of cancer patients and/or an association between elevated serum CCL2 and poor prognosis [41–51]. Other studies, however, found either no association between the serum CCL2 levels of cancer patients and clinical variables or that lower serum CCL2 levels were associated with poor prognosis or that higher serum CCL2 levels were associated with favorable prognosis [52–60].

Whether the disparate findings of the studies cited above are due to differences in tumor stage, CCL2 being associated with a tumorigenic response in some cases and to a tumoricidal response in others, differing immune suppression mechanisms in different tumor types or the patient cohorts studied, or to some other factor is not known. It is clear, however, that the role of CCL2 in tumorigenesis is likely to be affected by tumor-specific factors. The current study was undertaken to investigate serum CCL2 levels in mesothelioma patients. We found that serum CCL2 levels were increased in mesothelioma patients and that this increase was dependent on advancing mesothelioma stage.

## Methods

### Subjects

Healthy, unexposed volunteers (41 volunteers; 10 females and 31 males; age 56 ± 20.0 years; Range 23–91 years): Serum samples were collected from teaching and research staff at the Nagoya City University Graduate School of Medical Sciences and residents/patients at Nogoyashi Koseiin Medical Welfare Center Hospital (Koseiin Hospital). These subjects had no history of exposure to asbestos and were free from lung and pleural lesions on periodical (once or twice a year) institutional health examinations.

Healthy subjects possibly exposed to asbestos (356 subjects; 33 females and 323 males; age 68.7 ± 8.3 years; Range 35–96 years): Serum samples were collected from patients who visited or were hospitalized in the Japan Labour Health and Welfare Organization Asahi Rosai Hospital and the Saiseikai Chuwa Hospital. All of the enrolled subjects possibly exposed to asbestos had certified documents issued by the Japanese Ministry of Health, Labour and Welfare for the compensation of medical care. These subjects had no detectable asbestos-associated disease. Since the hospital records of patients not suffering from mesothelioma were not available to us, it is not known whether any of the subjects in this group had a health condition or treatment that would increase their serum CCL2 levels, for example see patient 356 (Additional file 1: Table S1). However, while there was a tendency for this group to have higher serum CCL2 levels compared to the healthy, unexposed volunteers, the difference between these groups was not statistically significant.

Mesothelioma patients (50 patients; 5 females and 45 males; age 72.5 ± 8.6 years; Range 57–99 years): Serum samples were collected from patients who were hospitalized in the Okayama Rosai Hospital, Asahi Rosai Hospital, Saiseikai Chuwa Hospital, Daido Hospital, and Nagoya City University Hospital. The diagnosis of MPM was made by biopsy examination combined with chest computed tomography examinations. Histological types of MPM were sarcomatoid, epithelioid, and biphasic.

All participants were provided written informed consent before inclusion in the study. Serum samples were then obtained, coded, and stored in aliquots at − 80 °C until use.

### Assay method

Enzyme-linked immune-absorbent assay (ELISA) kits (CCL2: DCP00, R&D systems, Minneapolis, USA) were used for measuring CCL2, following the manufacturer’s instructions. The minimum detectable level of human CCL2 ranged between 0.57 and 10.0 pg/ml for these ELISA kits. All samples had measured CCL2 levels above the minimum detectable levels.

### Statistics

In Table 1, patient age and serum CCL2 levels are presented as mean ± SD. In Tables 2, 3, 4 and 5, Analysis of Variance (ANOVA) was used to calculate the estimated marginal means and standard errors. Fisher’s exact test was used to test the significance of the differences of the nominal data (the data pertaining to gender). The Kruskal-Wallis (one-way ANOVA) test was used to test the significance of the differences in patient age. Analysis of covariance (ANCOVA) was used to compare the estimated marginal means of serum CCL2 levels adjusted for the covariates of age and gender. The homogeneity of the variance of the serum CCL2 levels was tested using Welch’s test. The significance of the differences between the means was tested using the Bonferroni test when the variance was homogenous and Tamhane’s T2 test when the variance was not homogenous. *p*-values were determined using pairwise comparison tests (pairwise comparisons are shown in Additional file 3: Tables S3 - S8). *p*-values < 0.05 were considered statistically significant. All statistical analyses were carried out with statistical software package SPSS 24.0 (SPSS, Chicago, IL, USA).

**Table 1:** Gender, age, and serum CCL2 levels of the study subjects. (Individual patient data is shown in Additional file 1: Table S1)

	Number of Patients	Gender		Age	Serum CCL2 (pg/ml)
		Women	Men		
Unexposed (no apparent disease)	41	10	31	56.0 ± 20.0	275.2 ± 98.2
Possibly Exposed (no apparent disease)	356	33	323	68.7 ± 8.3	307.5 ± 117.7
Mesothelioma (all patients)	50	5	45	72.5 ± 8.6	421.3 ± 295.1^a,b^
Mesothelioma (stage 1 patients)	12	0	12	72.8 ± 9.1	289.9 ± 115.4
Mesothelioma (stage 2 patients)	5	0	5	75.6 ± 7.1	281.0 ± 111.2
Mesothelioma (stage 3 patients)	14	1	13	74.3 ± 10.7	486.0 ± 333.4^c,d^
Mesothelioma (stage 4 patients)	19	4	15	70.2 ± 6.8	493.5 ± 346.7^c,d^

^a^Different from the Unexposed (no apparent disease) group at *p* < 0.01

^b^Different from the Possibly Exposed (no apparent disease) group at *p* < 0.05

^c^Different from the Unexposed (no apparent disease) and the Possibly Exposed (no apparent disease) groups at *p* < 0.001

^d^Different from the Mesothelioma (stage 1 patients) group at *p* < 0.01

**Table 2:** Serum CCL2 levels of the study subjects after adjusting the data for the covariates of gender and age

	Unadjusted Data (ANOVA)				Adjusted Data (ANCOVA)			
	Serum CCL2 (pg/ml)	Std Error	95% CI		Estimated Serum CCL2 (pg/ml)	Std Error	95% CI	
			Lower Limit	Upper Limit			Lower Limit	Upper Limit
Unexposed (no apparent disease)	275.2	22.9	230.2	320.3	303.5	24.2	256.0	351.1
Possibly Exposed (no apparent disease)	307.5	7.8	292.2	322.8	305.6	7.7	290.4	320.8
Mesothelioma (all patients)	421.3^a,c^	20.8	380.5	462.1	411.8^b,c^	20.8	370.9	452.6

^a^Different from the Unexposed (no apparent disease) group at *p* < 0.001

^b^Different from the Unexposed (no apparent disease) group at *p* < 0.01

^c^Different from the Possibly Exposed (no apparent disease) group at *p* < 0.001

**Table 3:** Serum CCL2 levels of the study subjects after adjusting the data for the covariates of gender and age

	Unadjusted Data (ANOVA)				Adjusted Data (ANCOVA)			
	Serum CCL2 (pg/ml)	Std Error	95% CI		Estimated Serum CCL2 (pg/ml)	Std Error	95% CI	
			Lower Limit	Upper Limit			Lower Limit	Upper Limit
Unexposed (no apparent disease)	275.2	22.9	230.2	320.3	305.5	24.2	256.0	351.1
Possibly Exposed (no apparent disease)	307.5	7.8	292.2	322.8	305.4	7.7	290.4	320.8
Mesothelioma (stage 1 patients)	289.9	41.5	208.4	371.4	275.7	41.1	195.0	356.5
Mesothelioma (stage 2 patients)	281.0	64.4	154.7	407.3	261.0	63.6	136.0	386.0
Mesothelioma (stage 3 patients)	486.0^a,c,d^	38.4	410.5	561.5	471.4^b,c,d^	38.1	396.4	546.3
Mesothelioma (stage 4 patients)	493.5^a,c,d^	33.0	428.7	558.3	492.5^a,c,d,e^	32.7	428.3	556.7

^a^Different from the Unexposed (no apparent disease) group at *p* < 0.001

^b^Different from the Unexposed (no apparent disease) group at *p* < 0.01

^c^Different from the Possibly Exposed (no apparent disease) group at *p* < 0.001

^d^Different from the Mesothelioma stage 1 patients group at *p* < 0.01

^e^Different from the Mesothelioma stage 2 patients group at *p* < 0.05

**Table 4:** Serum CCL2 levels of the study subjects after removing patients 31 and 50 and adjusting the data for the covariates of gender and age

	Unadjusted Data (ANOVA)				Adjusted Data (ANCOVA)			
	Serum CCL2 (pg/ml)	Std Error	95% CI		Estimated Serum CCL2 (pg/ml)	Std Error	95% CI	
			Lower Limit	Upper Limit			Lower Limit	Upper Limit
Unexposed (no apparent disease)	275.2	18.5	238.9	311.6	308.6	19.3	270.7	346.4
Possibly Exposed (no apparent disease)	307.5	6.3	295.2	319.9	305.4	6.1	293.3	317.4
Mesothelioma (all patients)	368.5^a^	17.1	334.9	402.1	356.0^b^	16.9	322.8	389.2

^a^Different from the Unexposed (no apparent disease) and the Possibly Exposed (no apparent disease) groups at *p* < 0.01

^b^Different from the Possibly Exposed (no apparent disease) groups at *p* < 0.05

**Table 5:** Serum CCL2 levels of the study subjects after removing patients 31 and 50 and adjusting the data for the covariates of gender and age

	Unadjusted Data (ANOVA)				Adjusted Data (ANCOVA)			
	Serum CCL2 (pg/ml)	Std Error	95% CI		Estimated Serum CCL2 (pg/ml)	Std Error	95% CI	
			Lower Limit	Upper Limit			Lower Limit	Upper Limit
Unexposed (no apparent disease)	275.2	18.3	239.3	311.2	305.5	24.2	256.0	351.1
Possibly Exposed (no apparent disease)	307.5	6.2	295.3	319.7	305.4	7.7	290.4	320.8
Mesothelioma (stage 1 patients)	289.9	33.8	223.5	356.4	275.7	41.1	195.0	356.5
Mesothelioma (stage 2 patients)	281.0	52.4	178.1	383.9	261.0	63.6	136.0	386.0
Mesothelioma (stage 3 patients)	402.7^b^	32.5	338.9	466.5	471.4	38.1	396.4	546.3
Mesothelioma (stage 4 patients)	420.5^a,c,d^	27.6	366.3	474.8	492.5^b,c,d^	32.7	428.3	556.7

^a^Different from the Unexposed (no apparent disease) group at *p* < 0.001

^b^Different from the Unexposed (no apparent disease) group at *p* < 0.05

^c^Different from the Possibly Exposed (no apparent disease) group at *p* < 0.01

^d^Different from the Mesothelioma stage 1 patients group at *p* < 0.05

## Results

A summary of the gender, age, and serum CCL2 levels of the study subjects is shown in Table 1. Individual CCL2 levels are shown in Additional file 1: Table S1. The pairwise comparisons of the groups is shown in Additional file 3: Tables S3 and S4. The mean CCL2 level in the serum of the mesothelioma patients is significantly elevated compared to the Possibly Exposed (no apparent disease) group, and this increase is dependent on the stage of the disease.

It is known that serum CCL2 levels increase with age [61–63], and as can be seen in Table 1 the mean CCL2 level in the serum of the Possibly Exposed (no apparent disease) group, age 68.7 ± 8.3 yrs., is higher than that of the Unexposed (no apparent disease) group, age 56.0 ± 20.0 yrs.: the age ranges of the study participants are shown in Additional file 2: Table S2. Analysis of the age of the patients using the Kruskal-Wallis (one-way ANOVA) test shows an age difference between the patients in the different groups (*p* < 0.05). Fisher’s exact test also shows a gender difference between groups (*p* < 0.05): see Methods for the gender of the study participants. Therefore, the data was re-analyzed based on covariates of age (67.97) and gender (1.11). In Tables 2, 3, 4 and 5, Analysis of Variance (ANOVA) was used to calculate the estimated marginal means and standard error. Subsequently, Analysis of covariance (ANCOVA) was used to compare the estimated marginal means adjusted for covariates of age and gender.

Tables 2 and 3 show the unadjusted serum CCL2 means and 95% confidence intervals and the estimated CCL2 means and 95% confidence intervals when the data is adjusted based on the covariates of age and gender. In Table 2, the data was adjusted using the Unexposed (no apparent disease), Possibly Exposed (no apparent disease), and Mesothelioma (all patients) groups. The pairwise comparisons of these groups is shown in Additional file 3: Table S5. In Table 3, the data was adjusted using the Unexposed (no apparent disease), Possibly Exposed (no apparent disease), and Mesothelioma stages 1–4 groups. The pairwise comparisons of these groups is shown in Additional file 3: Table S6. After adjusting the data, the estimated mean CCL2 level in the serum of the mesothelioma patients is significantly elevated compared to the Possibly Exposed (no apparent disease) group, and this increase is dependent on the stage of the disease.

Two patients in the Mesothelioma group, patients 31 and 50 (Additional file 1: Table S1), had extraordinarily high levels of serum CCL2. Removal of these two patients reduces the serum CCL2 levels in the mesothelioma all patients, stage 3 patients, and stage 4 patients groups to 368.5 ± 138.1, 402.7 ± 123.2, and 420.5 ± 141.9, respectively. Tables 4 and 5 show the results when these two patients are removed from data analysis. Table 4 shows the unadjusted serum CCL2 means and 95% confidence intervals and the estimated CCL2 means and 95% confidence intervals when the data is adjusted based on the covariates of age and gender using the Unexposed (no apparent disease), Possibly Exposed (no apparent disease), and Mesothelioma (all patients) groups. The pairwise comparisons of these groups is shown in Additional file 3: Table S7. Table 5 shows the unadjusted serum CCL2 means and 95% confidence intervals and the estimated CCL2 means and 95% confidence intervals when the data is adjusted based on the covariates of age and gender using the Unexposed (no apparent disease), Possibly Exposed (no apparent disease), and Mesothelioma stages 1–4 groups. The pairwise comparisons of these groups is shown in Additional file 3: Table S8. After removal of patients 31 and 50 from the data analysis, CCL2 levels in the mesothelioma patients are still significantly higher than the CCL2 levels in the Unexposed (no apparent disease) and the Possibly Exposed (no apparent disease) groups, and this increase is dependent on the stage of the disease.

## Discussion

In this study we measured the levels of CCL2 in the serum of 41 healthy volunteers who have not been exposed to asbestos, 356 healthy subjects who have possibly been exposed to asbestos, and 50 mesothelioma patients. The mean CCL2 level in the serum of the mesothelioma patients was significantly elevated compared to both the healthy volunteers who have not been exposed to asbestos and the healthy subjects who have possibly been exposed to asbestos (see Table 1). However, it is known that serum CCL2 levels increase with normal aging [61–63], and analysis of the age of the patients using the Kruskal-Wallis (one-way ANOVA) test showed an age difference between the patients in the Possibly Exposed (no apparent disease) and the mesothelioma groups. Fisher’s exact test also showed a gender difference between these groups. Therefore, the data was re-analyzed based on covariates of age (67.97) and gender (1.11). Re-analysis of the data after adjusting for age and gender did not change the conclusions of the study: serum CCL2 was elevated in mesothelioma patients (see Table 2). Mesothelioma patients 31 and 50 (see Additional file 1: Table S1) had exceptionally high levels of CCL2. After removal of these two patients’ data from analysis, serum CCL2 was still elevated in mesothelioma patients (see Table 4). Therefore, our data indicate that serum CCL2 levels were increased in mesothelioma patients and this increase was not dependent on the age of the patients in the Mesothelioma group or on the presence of the two patients in the Mesothelioma group with exceptionally high levels of serum CCL2. Elevated CCL2 in the serum of mesothelioma patients is in agreement with the high levels of CCL2 present in the pleural effusions of mesothelioma patients reported by Gueugnon et al. [64].

The increase in the serum levels of CCL2 in the mesothelioma patients was dependent on the stage of the disease (see Table 1). Reanalysis of the data adjusting for age and gender also indicated elevated levels of serum CCL2 depended on mesothelioma stage (see Tables 2 and 3). The dependence on mesothelioma stage was still apparent after removal of the two mesothelioma patients with exceptionally high levels of serum CCL2 from data analysis (see Tables 4 and 5). Therefore, as with the increase in the levels of CCL2 in the serum of mesothelioma patients, the dependence of this increase on disease stage was not due to the age of the patients in the Mesothelioma group or on the presence of the two patients in the Mesothelioma group with exceptionally high levels of serum CCL2.

The mean CCL2 level in the serum of the healthy subjects who have possibly been exposed to asbestos was elevated compared to the healthy volunteers who have not been exposed to asbestos. However, as noted above, it is known that serum CCL2 levels increase during normal ageing [61–63]. Thus, the levels of CCL2 in the serum in these two groups followed the expected pattern, lower in the healthy unexposed group consisting of primarily younger patients and higher in the healthy possibly exposed group consisting of primarily older patients.

Several studies have reported that increased expression of CCL2 in tumor tissue is associated with advanced tumor stage and worse prognosis: These studies include patients with breast cancer [65–68], prostate cancer [69, 70], gastric cancer [71], colorectal cancer [72, 73], esophageal squamous cell carcinoma [74], head and neck squamous cell carcinoma [75], and glial tumors [47]. In agreement with these findings, a number of studies report elevated levels of CCL2 in the serum of cancer patients and/or an association between elevated serum CCL2 and poor prognosis: Moogooei et al. [47] and Pan et al. [48] report elevated levels of serum CCL2 in patients with glial tumors and lung cancer. Lu et al. [45] and Sharma et al. [49] report an association between elevated serum CCL2 levels and poor prognosis in patients with prostate cancer, and Lu et al. [44] report an association between elevated serum CCL2 levels and poor prognosis in patients with nasopharyngeal cancer. Cai et al. [41], Wang et al. [50], Wu et al. [51], Lubowicka et al. [46], and Hefler et al. [42] report elevated levels of serum CCL2 in patients with lung, liver, gastric, breast, and ovarian cancer and that increased serum CCL2 was associated with poor prognosis. Lebrecht et al. [43] did not find a difference in serum CCL2 levels between breast cancer patients and normal donors, but they did find an association between serum CCL2 and poor prognosis.

However, there are also reports that increased expression of CCL2 in tumor tissue is associated with better prognosis: These studies include patients with gastric cancer [59], colorectal cancer [76], liver cancer [77], and non-small cell lung cancer [78]. There are also a number of studies, that report either that serum CCL2 levels in cancer patients are not related to clinical variables or that higher serum CCL2 levels are associated with a better prognosis and/or that lower serum CCL2 levels are associated with worse prognosis. Tas et al. [58], Tsaur et al. [60], and Monti et al. [56] found elevated serum CCL2 levels in patients with gastric, prostate, and pancreas cancer. However, Tas et al. report that while gastric cancer patients who responded to chemotherapy had lower serum CCL2 than non-responders, there was no association between serum CCL2 and any measured clinical variables; Tsuar et al. report that elevated serum CCL2 was negatively correlated with PSA value in prostate cancer patients; and Monti et al. report that elevated serum CCL2 was associated with increased survival in pancreas cancer patients. Farren et al. [54] also report that elevated serum CCL2 levels correlated with increased survival in pancreas cancer patients. Sullivan et al. [57] report that there was no difference in serum CCL2 levels between pancreas cancer patients and normal donors and that serum CCL2 did not correlate with any measured clinico-pathological parameters. Koper et al. [55], Ding et al. [53], and Tonouchi et al. [59] report that serum CCL2 levels were decreased in patients with astrocytic brain tumors, oral squamous cell carcinoma, and gastric cancer, and Tonouchi et al. report CCL2 levels tended to decrease in accordance with disease progression and that decreased serum CCL2 levels were associated with poor survival. Dehqanzada et al. [52] report that elevated serum CCL2 levels correlated with favorable prognostic variables in patients with breast cancer.

Thus, the association between serum CCL2 levels and different cancers appears to be variable. Since mesotheliomas are heavily infiltrated by macrophages [7–10] and likely to be infiltrated by MDSCs [33, 34], our finding that CCL2 is elevated in the serum of patients with advanced mesothelioma is consistent with a disease in which the CCL2/CCR2 axis and myeloid-derived cells play an important part. Consequently, therapies that prove effective against other cancers in which the CCL2/CCR2 axis and myeloid-derived cells are associated with disease progression may also prove effective with mesothelioma patients. There is considerable interest in developing therapies that target CCL2/CCR2 and tumor-resident myeloid cells [5, 22, 79–85]. Numerous clinical trials employing these therapies as part of the treatment regimen have been carried out or are currently being pursued [86–94]. The success or failure of these trials will have important implications for the treatment of mesothelioma. Another aspect of increased CCL2 in the serum of mesothelioma patients is that it may be possible to use serum CCL2 to monitor a patient’s response to treatment [95].

## Conclusions

CCL2 levels are elevated in mesothelioma patients and the increase is dependent on the stage of the disease. This is consistent with the premise that the CCL2/CCR2 axis and myeloid-derived cells play an important role in mesothelioma and disease progression. Other types of cancer also cause stage-dependent increases in serum CCL2. Therapies are being developed that target CCL2/CCR2 and tumor resident myeloid cells, and clinical trials are being pursued that use these therapies as part of the treatment regimen. The results of trials with patients with a similar pattern of CCL2 as mesothelioma patients will have important implications for the treatment of mesothelioma.

## Supplementary information

**Additional file 1: Table S1.** Serum CCL2 levels: Individual patient data.

**Additional file 2: Table S2.** Age of the study participants.

**Additional file 3: Table S3.** Pairwise comparisons of the Unexposed_No apparent disease, Possibly Exposed_no apparent disease, and Mesothelioma Patients groups. **Table S4.** Pairwise comparisons of the Unexposed_no apparent disease, Possibly Exposed_no apparent disease, and Mesothelioma Stage 1–4 groups. **Table S5.** Pairwise comparisons of the Unexposed_no apparent disease, Possibly Exposed_no apparent disease, and Mesothelioma Patients groups. **Table S6.** Pairwise comparisons of the Unexposed_no apparent disease, Possibly Exposed_no apparent disease, and Mesothelioma Stages 1–4 groups. **Table S7.** Pairwise comparisons of the Unexposed_no apparent disease, Possibly Exposed_no apparent disease, and Mesothelioma Patients groups, with patients 31 and 50 removed from data analysis. **Table S8.** Pairwise comparisons of the Unexposed_no apparent disease, Possibly Exposed_no apparent disease, and Mesothelioma Stages 1–4 groups, with patients 31 and 50 removed from data analysis.

## Data Availability

All data is available in Addition file S1.

## Abbreviations

CCL2: C-C motif chemokine ligand 2
MCP-1: Monocyte chemotactic protein-1
MDSC: Myeloid-derived suppressor cell
MPM: Malignant pleural mesothelioma
