# Supplementary material for: Serum levels of the chemokine CCL2 are elevated in malignant pleural mesothelioma patients
Source: BMC Cancer. 2019 Dec 10;19:1204. doi: 10.1186/s12885-019-6419-1 (PMC6905076; doi:10.1186/s12885-019-6419-1)
Supplement: Supplementary file 1 — Additional file 1: Table S1. Serum CCL2 levels: Individual patient data. [file 12885_2019_6419_MOESM1_ESM.pdf]

Serum levels of the chemokine CCL2 are elevated in malignant pleural mesothelioma patients

Takumi Kishimoto[1,*], Nobukazu Fujimoto[1], Takeshi Ebara[2], Toyonori Omori[3], Tetsuya Oguri[4], Akio Niimi[4], Takako Yokoyama[5], Munehiro Kato[5], Ikuji Usami[5], Masayuki Nishio[6], Kosho Yoshikawa[6], Takeshi Tokuyama[7], Mouka Tamura[8], Ken Tsuboi[9], Yoichi Matsuo[9], Jiegou Xu[10,11], Satoru Takahashi[12], Mohamed Abdelgied[11,12,13], William T. Alexander[11], David B. Alexander[11,*] and Hiroyuki Tsuda[11,*]

1 Japan Organization of Occupational Health and Safety, Research Center for Asbestos-related Diseases, Okayama Rosai Hospital. Okayama, Japan
2 Department of Occupational and Environmental Health, Nagoya City University Graduate School of Medical Sciences. Nagoya, Japan
3 Department of Healthcare Policy and Management, Nagoya City University Graduate School of Medical Sciences. Nagoya, Japan
4 Department of Respiratory Medicine, Allergy and Clinical Immunology, Nagoya City University Graduate School of Medical Sciences. Nagoya Japan
5 Japan Organization of Occupational Health and Safety, Department of Respiratory Medicine, Asahi Rosai Hospital. Owariasahi Japan
6 Department of Respiratory Medicine, Daido Hospital. Nagoya, Japan
7 Department of Internal Medicine, Saiseikai Chuwa Hospital. Sakurai, Nara, Japan
8 Department of Internal Medicine, National Hospital Organization Nara Medical Center. Nara, Japan
9 Department of Gastroenterological Surgery, Nagoya City University Graduate School of Medical Sciences. Nagoya, Japan
10 Department of Immunology, College of Basic Medical Sciences, Anhui Medical University. Hefei, China
11 Nanotoxicology Project, Nagoya City University. Nagoya, Japan
12 Department of Experimental Pathology and Tumor Biology, Nagoya City University Graduate School of Medical Sciences. Nagoya, Japan
13 Department of Forensic Medicine and Toxicology, Faculty of Veterinary Medicine. Beni-Suef University. Beni-Suef, Egypt.

***Correspondence:** nakisimt@okayamah.johas.go.jp, dalexand@phar.nagoya-cu.ac.jp, htsuda@phar.nagoya-cu.ac.jp

Table S1. Serum CCL2 levels: Individual patient data.

Stage 0: These subjects had no apparent disease at the time of blood collection.

Unexposed No Disease Patient Code	Stage	CCL2 (pg/ml)
1	0	146
2	0	160
3	0	176
4	0	177
5	0	177
6	0	182
7	0	188
8	0	190
9	0	193
10	0	198
11	0	198
12	0	201
13	0	203
14	0	206
15	0	207
16	0	211
17	0	220
18	0	223
19	0	237
20	0	242
21	0	250
22	0	251
23	0	262
24	0	265
25	0	280
26	0	302
27	0	303
28	0	305
29	0	316
30	0	321
31	0	323
32	0	326
33	0	326
34	0	331
35	0	403
36	0	426
37	0	456
38	0	461
39	0	475
40	0	478
41	0	490

Exposed No Disease Patient Code	Stage	CCL2 (pg/ml)
1	0	77
2	0	93
3	0	96
4	0	104
5	0	122
6	0	128
7	0	135
8	0	143
9	0	143
10	0	144
11	0	146
12	0	150
13	0	152
14	0	156
15	0	161
16	0	161
17	0	161
18	0	162
19	0	163
20	0	164
21	0	165
22	0	168
23	0	169
24	0	171
25	0	175
26	0	176
27	0	181
28	0	182
29	0	182
30	0	183
31	0	188
32	0	190
33	0	192
34	0	193
35	0	193
36	0	194
37	0	194
38	0	195
39	0	195
40	0	197
41	0	198
42	0	199
43	0	202
44	0	202
45	0	203
46	0	203
47	0	204
48	0	209
49	0	210
50	0	211
51	0	211
52	0	213
53	0	213
54	0	214
55	0	215
56	0	215

Mesothelioma Patient Code	Stage	CCL2 (pg/ml)
1	1	119
2	1	194
3	1	225
4	1	232
5	1	238
6	1	245
7	1	272
8	1	287
9	1	335
10	1	343
11	1	436
12	1	553
13	2	120
14	2	259
15	2	264
16	2	344
17	2	418
18	3	168
19	3	230
20	3	315
21	3	335
22	3	391
23	3	415
24	3	415
25	3	423
26	3	438
27	3	478
28	3	486
29	3	487
30	3	654
31	3	1569
32	4	181
33	4	217
34	4	233
35	4	293
36	4	300
37	4	337
38	4	358
39	4	388
40	4	421
41	4	429
42	4	464
43	4	498
44	4	516
45	4	529
46	4	534
47	4	616
48	4	625
49	4	630
50	4	1807

57	0	215
58	0	216
59	0	216
60	0	216
61	0	217
62	0	218
63	0	220
64	0	220
65	0	221
66	0	222
67	0	224
68	0	226
69	0	227
70	0	227
71	0	228
72	0	229
73	0	231
74	0	232
75	0	234
76	0	234
77	0	235
78	0	237
79	0	237
80	0	240
81	0	241
82	0	241
83	0	241
84	0	242
85	0	242
86	0	243
87	0	244
88	0	244
89	0	245
90	0	245
91	0	246
92	0	248
93	0	248
94	0	250
95	0	250
96	0	251
97	0	252
98	0	254
99	0	254
100	0	254
101	0	254
102	0	255
103	0	255
104	0	256
105	0	256
106	0	256
107	0	257
108	0	257
109	0	257
110	0	257
111	0	257
112	0	257
113	0	257
114	0	258
115	0	258
116	0	259
117	0	260
118	0	262
119	0	262
120	0	262

121	0	262
122	0	263
123	0	263
124	0	263
125	0	264
126	0	264
127	0	265
128	0	265
129	0	267
130	0	267
131	0	267
132	0	269
133	0	269
134	0	269
135	0	269
136	0	270
137	0	270
138	0	270
139	0	270
140	0	271
141	0	271
142	0	272
143	0	272
144	0	273
145	0	275
146	0	275
147	0	277
148	0	277
149	0	278
150	0	281
151	0	281
152	0	281
153	0	282
154	0	283
155	0	283
156	0	283
157	0	283
158	0	283
159	0	283
160	0	284
161	0	285
162	0	285
163	0	285
164	0	286
165	0	287
166	0	287
167	0	288
168	0	289
169	0	289
170	0	290
171	0	290
172	0	291
173	0	291
174	0	291
175	0	292
176	0	292
177	0	292
178	0	293
179	0	295
180	0	295
181	0	296
182	0	298
183	0	298
184	0	298

185	0	298
186	0	298
187	0	300
188	0	300
189	0	300
190	0	301
191	0	301
192	0	301
193	0	302
194	0	302
195	0	302
196	0	303
197	0	304
198	0	305
199	0	306
200	0	306
201	0	307
202	0	307
203	0	308
204	0	308
205	0	308
206	0	308
207	0	309
208	0	310
209	0	310
210	0	310
211	0	310
212	0	311
213	0	311
214	0	311
215	0	312
216	0	313
217	0	313
218	0	314
219	0	316
220	0	317
221	0	318
222	0	318
223	0	318
224	0	319
225	0	319
226	0	323
227	0	323
228	0	323
229	0	323
230	0	324
231	0	324
232	0	326
233	0	327
234	0	329
235	0	329
236	0	330
237	0	330
238	0	331
239	0	332
240	0	332
241	0	333
242	0	334
243	0	335
244	0	335
245	0	335
246	0	335
247	0	336
248	0	336

249	0	337
250	0	339
251	0	340
252	0	342
253	0	344
254	0	344
255	0	348
256	0	348
257	0	349
258	0	349
259	0	349
260	0	350
261	0	351
262	0	352
263	0	354
264	0	355
265	0	359
266	0	361
267	0	361
268	0	362
269	0	362
270	0	363
271	0	364
272	0	365
273	0	366
274	0	366
275	0	367
276	0	367
277	0	368
278	0	369
279	0	371
280	0	375
281	0	378
282	0	379
283	0	380
284	0	381
285	0	382
286	0	384
287	0	385
288	0	385
289	0	386
290	0	386
291	0	388
292	0	389
293	0	389
294	0	390
295	0	390
296	0	393
297	0	393
298	0	394
299	0	395
300	0	395
301	0	395
302	0	396
303	0	397
304	0	397
305	0	400
306	0	402
307	0	405
308	0	405
309	0	406
310	0	406
311	0	407
312	0	408

313	0	409
314	0	410
315	0	411
316	0	414
317	0	419
318	0	421
319	0	421
320	0	423
321	0	424
322	0	435
323	0	435
324	0	436
325	0	438
326	0	439
327	0	442
328	0	444
329	0	450
330	0	453
331	0	455
332	0	456
333	0	458
334	0	459
335	0	462
336	0	466
337	0	468
338	0	471
339	0	476
340	0	476
341	0	479
342	0	484
343	0	494
344	0	494
345	0	513
346	0	520
347	0	525
348	0	529
349	0	537
350	0	553
351	0	557
352	0	558
353	0	574
354	0	592
355	0	690
356	0	1658
