# Supplementary material for: Serum levels of the chemokine CCL2 are elevated in malignant pleural mesothelioma patients
Source: BMC Cancer. 2019 Dec 10;19:1204. doi: 10.1186/s12885-019-6419-1 (PMC6905076; doi:10.1186/s12885-019-6419-1)
Supplement: Supplementary file 2 — Additional file 2: Table S2. Age of the study participants. [file 12885_2019_6419_MOESM2_ESM.pdf]

Serum levels of the chemokine CCL2 are elevated in malignant pleural mesothelioma patients

Takumi Kishimoto[1,*], Nobukazu Fujimoto[1], Takeshi Ebara[2], Toyonori Omori[3], Tetsuya Oguri[4], Akio Niimi[4], Takako Yokoyama[5], Munehiro Kato[5], Ikuji Usami[5], Masayuki Nishio[6], Kosho Yoshikawa[6], Takeshi Tokuyama[7], Mouka Tamura[8], Ken Tsuboi[9], Yoichi Matsuo[9], Jiegou Xu[10,11], Satoru Takahashi[12], Mohamed Abdelgied[11,12,13], William T. Alexander[11], David B. Alexander[11,*] and Hiroyuki Tsuda[11,*]

1 Japan Organization of Occupational Health and Safety, Research Center for Asbestos-related Diseases, Okayama Rosai Hospital. Okayama, Japan

2 Department of Occupational and Environmental Health, Nagoya City University Graduate School of Medical Sciences. Nagoya, Japan

3 Department of Healthcare Policy and Management, Nagoya City University Graduate School of Medical Sciences. Nagoya, Japan

4 Department of Respiratory Medicine, Allergy and Clinical Immunology, Nagoya City University Graduate School of Medical Sciences. Nagoya Japan

5 Japan Organization of Occupational Health and Safety, Department of Respiratory Medicine, Asahi Rosai Hospital. Owariasahi Japan

6 Department of Respiratory Medicine, Daido Hospital. Nagoya, Japan

7 Department of Internal Medicine, Saiseikai Chuwa Hospital. Sakurai, Nara, Japan

8 Department of Internal Medicine, National Hospital Organization Nara Medical Center. Nara, Japan

9 Department of Gastroenterological Surgery, Nagoya City University Graduate School of Medical Sciences. Nagoya, Japan

10 Department of Immunology, College of Basic Medical Sciences, Anhui Medical University. Hefei, China

11 Nanotoxicology Project, Nagoya City University. Nagoya, Japan

12 Department of Experimental Pathology and Tumor Biology, Nagoya City University Graduate School of Medical Sciences. Nagoya, Japan

13 Department of Forensic Medicine and Toxicology, Faculty of Veterinary Medicine. Beni-Suef University. Beni-Suef, Egypt.

***Correspondence:** nakisimt@okayamah.johas.go.jp, dalexand@phar.nagoya-cu.ac.jp, htsuda@phar.nagoya-cu.ac.jp

Table S2. Age
Number of patients less than 25, 26-50, 51-75, and greater than 75 years of age.

Age (years)	Unexposed No Disease	Exposed No Disease	Mesothelioma Patients	Mesothelioma Stage 1	Mesothelioma Stage 2	Mesothelioma Stage 3	Mesothelioma Stage 4
< 25	3	0	0	0	0	0	0
26-50	14	15	0	0	0	0	0
51-75	15	274	35	8	2	9	16
>75	9	67	15	4	3	5	3
