# Supplementary material for: Serum levels of the chemokine CCL2 are elevated in malignant pleural mesothelioma patients
Source: BMC Cancer. 2019 Dec 10;19:1204. doi: 10.1186/s12885-019-6419-1 (PMC6905076; doi:10.1186/s12885-019-6419-1)
Supplement: Supplementary file 3 — Additional file 3: Table S3. Pairwise comparisons of the Unexposed_No apparent disease, Possibly Exposed_no apparent disease, and Mesothelioma Patients groups. Table S4. Pairwise comparisons of the Unexposed_no apparent disease, Possibly Exposed_no apparent disease, and Mesothelioma Stage 1–4 groups. Table S5. Pairwise comparisons of the Unexposed_no apparent disease, Possibly Exposed_no apparent disease, and Mesothelioma Patients groups. Table S6. Pairwise comparisons of the Unexposed_no apparent disease, Possibly Exposed_no apparent disease, and Mesothelioma Stages 1–4 groups. Table S7. Pairwise comparisons of the Unexposed_no apparent disease, Possibly Exposed_no apparent disease, and Mesothelioma Patients groups, with patients 31 and 50 removed from data analysis. Table S8. Pairwise comparisons of the Unexposed_no apparent disease, Possibly Exposed_no apparent disease, and Mesothelioma Stages 1–4 groups, with patients 31 and 50 removed from data analysis. [file 12885_2019_6419_MOESM3_ESM.pdf]

Serum levels of the chemokine CCL2 are elevated in malignant pleural mesothelioma patients

Takumi Kishimoto[1,*], Nobukazu Fujimoto[1], Takeshi Ebara[2], Toyonori Omori[3], Tetsuya Oguri[4], Akio Niimi[4], Takako Yokoyama[5], Munehiro Kato[5], Ikuji Usami[5], Masayuki Nishio[6], Kosho Yoshikawa[6], Takeshi Tokuyama[7], Mouka Tamura[8], Ken Tsuboi[9], Yoichi Matsuo[9], Jiegou Xu[10,11], Satoru Takahashi[12], Mohamed Abdelgied[11,12,13], William T. Alexander[11], David B. Alexander[11,*] and Hiroyuki Tsuda[11,*]

1 Japan Organization of Occupational Health and Safety, Research Center for Asbestos-related Diseases, Okayama Rosai Hospital. Okayama, Japan
2 Department of Occupational and Environmental Health, Nagoya City University Graduate School of Medical Sciences. Nagoya, Japan
3 Department of Healthcare Policy and Management, Nagoya City University Graduate School of Medical Sciences. Nagoya, Japan
4 Department of Respiratory Medicine, Allergy and Clinical Immunology, Nagoya City University Graduate School of Medical Sciences. Nagoya Japan
5 Japan Organization of Occupational Health and Safety, Department of Respiratory Medicine, Asahi Rosai Hospital. Owariasahi Japan
6 Department of Respiratory Medicine, Daido Hospital. Nagoya, Japan
7 Department of Internal Medicine, Saiseikai Chuwa Hospital. Sakurai, Nara, Japan
8 Department of Internal Medicine, National Hospital Organization Nara Medical Center. Nara, Japan
9 Department of Gastroenterological Surgery, Nagoya City University Graduate School of Medical Sciences. Nagoya, Japan
10 Department of Immunology, College of Basic Medical Sciences, Anhui Medical University. Hefei, China
11 Nanotoxicology Project, Nagoya City University. Nagoya, Japan
12 Department of Experimental Pathology and Tumor Biology, Nagoya City University Graduate School of Medical Sciences. Nagoya, Japan
13 Department of Forensic Medicine and Toxicology, Faculty of Veterinary Medicine. Beni-Suef University. Beni-Suef, Egypt.

***Correspondence:** nakisimt@okayamah.johas.go.jp, dalexand@phar.nagoya-cu.ac.jp, htsuda@phar.nagoya-cu.ac.jp

Table S3. Pairwise comparisons of the Unexposed_No apparent disease, Possibly Exposed_no apparent disease, and Mesothelioma Patients groups.

Category		Differences in the means	SE	p[a]	95% Confidence Interval Lower Limit	95% Confidence Interval Upper Limit
Unexposed no apparent disease	Possibly Exposed no apparent disease	-32.2898	16.5537	0.160	-73.075	8.496
	Mesothelioma	-146.0361	44.4644	0.005	-255.162	-36.910
Possibly Exposed no apparent disease	Unexposed no apparent disease	32.2898	16.5537	0.160	-8.496	73.075
	Mesothelioma	-113.7463	42.2000	0.028	-217.911	-9.582
Mesothelioma Patients	Unexposed no apparent disease	146.0361	44.4644	0.005	36.910	255.162
	Possibly Exposed no apparent disease	113.7463	42.2000	0.028	9.582	217.911

[a] These p-values are used in Table 1.

Welch's test indicated unequal variances in the Unexposed_No disease group, Possibly Exposed_no disease group, and Mesothelioma Patients group. Therefore, the Tamhane method was used for the pairwise comparisons of these groups.

Table S4. Pairwise comparisons of the Unexposed_no apparent disease, Possibly Exposed_no apparent disease, and Mesothelioma Stage 1-4 groups.

Category		Differences in the means	SE	p[b]	95% Confidence Interval	
					Lower Limit	Upper Limit
Unexposed no apparent disease	Possibly Exposed (nad)[a]	-32.2898	23.6974	1.000	-102.227	37.648
	Stage 1	-14.6728	47.1604	1.000	-153.856	124.511
	Stage 2	-5.7561	68.0650	1.000	-206.635	195.123
	Stage 3	-210.7561	44.4782	0.000	-342.024	-79.488
	Stage 4	-218.2298	39.8776	0.000	-335.920	-100.540
Possibly Exposed no apparent disease	Unexposed (nad)[a]	32.2898	23.6974	1.000	-37.648	102.227
	Stage 1	17.6170	42.1726	1.000	-106.846	142.080
	Stage 2	26.5337	64.7091	1.000	-164.441	217.508
	Stage 3	-178.4663	39.1502	0.000	-294.009	-62.923
	Stage 4	-185.9400	33.8326	0.000	-285.789	-86.090
Mesothelioma Stage 1	Unexposed (nad)[a]	14.6728	47.1604	1.000	-124.511	153.856
	Possibly Exposed (nad)[a]	-17.6170	42.1726	1.000	-142.080	106.846
	Stage 2	8.9167	76.4840	1.000	-216.809	234.642
	Stage 3	-196.0833	56.5267	0.009	-362.910	-29.257
	Stage 4	-203.5570	52.9828	0.002	-359.924	-47.190
Mesothelioma Stage 2	Unexposed (nad)[a]	5.7561	68.0650	1.000	-195.123	206.635
	Possibly Exposed (nad)[a]	-26.5337	64.7091	1.000	-217.508	164.441
	Stage 1	-8.9167	76.4840	1.000	-234.642	216.809
	Stage 3	-205.0000	74.8600	0.096	-425.933	15.933
	Stage 4	-212.4737	72.2213	0.051	-425.619	0.672
Mesothelioma Stage 3	Unexposed (nad)[a]	210.7561	44.4782	0.000	79.488	342.024
	Possibly Exposed (nad)[a]	178.4663*	39.1502	0.000	62.923	294.009
	Stage 1	196.0833	56.5267	0.009	29.257	362.910
	Stage 2	205.0000	74.8600	0.096	-15.933	425.933
	Stage 4	-7.4737	50.6102	1.000	-156.838	141.891
Mesothelioma Stage 4	Unexposed (nad)[a]	218.2298	39.8776	0.000	100.540	335.920
	Possibly Exposed (nad)[a]	185.9400	33.8326	0.000	86.090	285.789
	Stage 1	203.5570	52.9828	0.002	47.190	359.924
	Stage 2	212.4737	72.2213	0.051	-0.672	425.619
	Stage 3	7.4737	50.6102	1.000	-141.891	156.838

[a] no apparent disease

[b] These p-values are used in Table 1.

Welch's test indicated equal variances in the Unexposed_No disease group, Possibly Exposed_no disease group, and Mesothelioma stages 1-4 group. Therefore, the Bonferroni method was used for the pairwise comparisons of these groups.

Table S5. Pairwise comparisons of the Unexposed_no apparent disease, Possibly Exposed_no apparent disease, and Mesothelioma Patients groups.

		**Unadjusted Data (ANOVA)**					**Adjusted for covariates of gender and age (ANCOVA)**				
Category		Differences in the means	SE	p[a]	95% Confidence Interval Lower Limit	95% Confidence Interval Upper Limit	Differences in the means	SE	p[a]	95% Confidence Interval Lower Limit	95% Confidence Interval Upper Limit
Unexposed no apparent disease	Possibly Exposed no apparent disease	-32.290	24.210	0.549	-90.469	25.890	-2.080	25.587	1.000	-63.568	59.408
	Mesothelioma	-146.036	30.929	0.000	-220.361	-71.711	-108.219	32.694	0.003	-186.788	-29.650
Possibly Exposed no apparent disease	Unexposed no apparent disease	32.290	24.210	0.549	-25.890	90.469	2.080	25.587	1.000	-59.408	63.568
	Mesothelioma	-113.746	22.171	0.000	-167.024	-60.469	-106.139	22.104	0.000	-159.257	-53.021
Mesothelioma Patients	Unexposed no apparent disease	146.036	30.929	0.000	71.711	220.361	108.219	32.694	0.003	29.650	186.788
	Possibly Exposed no apparent disease	113.746	22.171	0.000	60.469	167.024	106.139	22.104	0.000	53.021	159.257

[a] These p-values are used in Table 2.

The estimated marginal means and the standard errors of the marginal means were calculated, and the Bonferroni method was used for pairwise comparison of the groups.

Table S6. Pairwise comparisons of the Unexposed_no apparent disease, Possibly Exposed_no apparent disease, and Mesothelioma Stages 1-4 groups.

Category		Unadjusted Data (ANOVA)					Adjusted for covariates of gender and age (ANCOVA)				
		Difference s in the means	SE	p[b]	95% Confidence Interval Lower Limit	95% Confidence Interval Upper Limit	Differences in the means	SE	p[b]	95% Confidence Interval Lower Limit	95% Confidence Interval Upper Limit
Unexposed no apparent disease	Exposed (nad)[a]	-32.2898	23.6974	1.000	-102.227	37.648	0.040	24.978	1.000	-73.680	73.760
	Stage 1	-14.6728	47.1604	1.000	-153.856	124.511	29.740	48.017	1.000	-111.975	171.455
	Stage 2	-5.7561	68.0650	1.000	-206.635	195.123	44.496	68.508	1.000	-157.694	246.687
	Stage 3	-210.7561	44.4782	0.000	-342.024	-79.488	-165.879[*]	45.619	0.005	-300.516	-31.242
	Stage 4	-218.2298	39.8776	0.000	-335.920	-100.540	-187.019[*]	40.509	0.000	-306.576	-67.463
Possibly Exposed no apparent disease	Unexposed (nad)[a]	32.2898	23.6974	1.000	-37.648	102.227	-0.040	24.978	1.000	-73.760	73.680
	Stage 1	17.6170	42.1726	1.000	-106.846	142.080	29.700	41.721	1.000	-93.433	152.834
	Stage 2	26.5337	64.7091	1.000	-164.441	217.508	44.457	64.010	1.000	-144.459	233.373
	Stage 3	-178.4663	39.1502	0.000	-294.009	-62.923	-165.918[*]	38.802	0.000	-280.437	-51.400
	Stage 4	-185.9400	33.8326	0.000	-285.789	-86.090	-187.059[*]	33.506	0.000	-285.946	-88.172
Mesothelioma Stage 1	Unexposed (nad)[a]	14.6728	47.1604	1.000	-124.511	153.856	-29.740	48.017	1.000	-171.455	111.975
	Exposed (nad)[a]	-17.6170	42.1726	1.000	-142.080	106.846	-29.700	41.721	1.000	-152.834	93.433
	Stage 2	8.9167	76.4840	1.000	-216.809	234.642	14.756	75.466	1.000	-207.970	237.483
	Stage 3	-196.0833	56.5267	0.009	-362.910	-29.257	-195.619[*]	55.793	0.008	-360.284	-30.953
	Stage 4	-203.5570	52.9828	0.002	-359.924	-47.190	-216.759[*]	52.474	0.001	-371.629	-61.889
Mesothelioma Stage 2	Unexposed (nad)[a]	5.7561	68.0650	1.000	-195.123	206.635	-44.496	68.508	1.000	-246.687	157.694
	Exposed (nad)[a]	-26.5337	64.7091	1.000	-217.508	164.441	-44.457	64.010	1.000	-233.373	144.459
	Stage 1	-8.9167	76.4840	1.000	-234.642	216.809	-14.756	75.466	1.000	-237.483	207.970
	Stage 3	-205.0000	74.8600	0.096	-425.933	15.933	-210.375	73.859	0.069	-428.360	7.609
	Stage 4	-212.4737	72.2213	0.051	-425.619	0.672	-231.516[*]	71.445	0.019	-442.376	-20.656
Mesothelioma Stage 3	Unexposed (nad)[a]	210.7561	44.4782	0.000	79.488	342.024	165.879[*]	45.619	0.005	31.242	300.516
	Exposed (nad)[a]	178.4663[*]	39.1502	0.000	62.923	294.009	165.918[*]	38.802	0.000	51.400	280.437
	Stage 1	196.0833	56.5267	0.009	29.257	362.910	195.619[*]	55.793	0.008	30.953	360.284
	Stage 2	205.0000	74.8600	0.096	-15.933	425.933	210.375	73.859	0.069	-7.609	428.360
	Stage 4	-7.4737	50.6102	1.000	-156.838	141.891	-21.141	50.065	1.000	-168.900	126.618
Mesothelioma Stage 4	Unexposed (nad)[a]	218.2298	39.8776	0.000	100.540	335.920	187.019[*]	40.509	0.000	67.463	306.576
	Exposed (nad)[a]	185.9400	33.8326	0.000	86.090	285.789	187.059[*]	33.506	0.000	88.172	285.946
	Stage 1	203.5570	52.9828	0.002	47.190	359.924	216.759[*]	52.474	0.001	61.889	371.629
	Stage 2	212.4737	72.2213	0.051	-0.672	425.619	231.516[*]	71.445	0.019	20.656	442.376
	Stage 3	7.4737	50.6102	1.000	-141.891	156.838	21.141	50.065	1.000	-126.618	168.900

[a] no apparent disease

[b] These p-values are used in Table 3.

The estimated marginal means and the standard errors of the marginal means were calculated, and the Bonferroni method was used for pairwise comparison of the groups.

Table S7. Pairwise comparisons of the Unexposed_no apparent disease, Possibly Exposed_no apparent disease, and Mesothelioma Patients groups, with patients 31 and 50 removed from data analysis.

		**Unadjusted Data (ANOVA)**					**Adjusted for covariates of gender and age (ANCOVA)**				
Category		Differences in the means	SE	p[a]	95% Confidence Interval		Differences in the means	SE	p[a]	95% Confidence Interval	
					Lower Limit	Upper Limit				Lower Limit	Upper Limit
Unexposed no apparent disease	Possibly Exposed no apparent disease	-32.290	19.528	0.297	-79.218	14.638	3.199	20.353	1.000	-45.712	52.110
	Mesothelioma	-93.256	25.180	0.001	-153.768	-32.745	-47.400	26.274	0.216	-110.540	15.740
Possibly Exposed no apparent disease	Unexposed no apparent disease	32.290	19.528	0.297	-14.638	79.218	-3.199	20.353	1.000	-52.110	45.712
	Mesothelioma	-60.966	18.206	0.003	-104.719	-17.214	-50.599*	17.915	0.015	-93.652	-7.546
Mesothelioma Patients	Unexposed no apparent disease	93.256	25.180	0.001	32.745	153.768	47.400	26.274	0.216	-15.740	110.540
	Possibly Exposed no apparent disease	60.966	18.206	0.003	17.214	104.719	50.599	17.915	0.015	7.546	93.652

[a] These p-values are used in Table 4.

The estimated marginal means and the standard errors of the marginal means were calculated, and the Bonferroni method was used for pairwise comparison of the groups.

Table S8. Pairwise comparisons of the Unexposed_no apparent disease, Possibly Exposed_no apparent disease, and Mesothelioma Stages 1-4 groups, with patients 31 and 50 removed from data analysis.

		Unadjusted Data (ANOVA)					Adjusted for covariates of gender and age (ANCOVA)				
Category		Differences in the means	SE	p[b]	95% Confidence Interval Lower Limit	95% Confidence Interval Upper Limit	Differences in the means	SE	p[b]	95% Confidence Interval Lower Limit	95% Confidence Interval Upper Limit
Unexposed no apparent disease	Exposed (nad)[a]	-32.290	19.314	1.000	-89.292	24.712	4.519	20.077	1.000	-54.736	63.775
	Stage 1	-14.673	38.437	1.000	-128.113	98.768	35.150	38.591	1.000	-78.749	149.049
	Stage 2	-5.756	55.475	1.000	-169.481	157.969	51.278	55.059	1.000	-111.225	213.780
	Stage 3	-127.448*	37.276	0.010	-237.462	-17.435	-75.459	37.636	0.684	-186.539	35.621
	Stage 4	-145.256*	33.112	0.000	-242.982	-47.530	-105.922*	33.233	0.023	-204.008	-7.836
Possibly Exposed no apparent disease	Unexposed (nad)[a]	32.290	19.314	1.000	-24.712	89.292	-4.519	20.077	1.000	-63.775	54.736
	Stage 1	17.617	34.372	1.000	-83.826	119.060	30.631	33.529	1.000	-68.329	129.590
	Stage 2	26.534	52.739	1.000	-129.119	182.187	46.758	51.442	1.000	-105.069	198.585
	Stage 3	-95.159	33.068	0.063	-192.754	2.437	-79.978	32.312	0.205	-175.346	15.389
	Stage 4	-112.966*	28.292	0.001	-196.467	-29.466	-110.441*	27.660	0.001	-192.079	-28.804
Mesothelioma Stage 1	Unexposed (nad)[a]	14.673	38.437	1.000	-98.768	128.113	-35.150	38.591	1.000	-149.049	78.749
	Exposed (nad)[a]	-17.617	34.372	1.000	-119.060	83.826	-30.631	33.529	1.000	-129.590	68.329
	Stage 2	8.917	62.336	1.000	-175.060	192.893	16.127	60.648	1.000	-162.871	195.126
	Stage 3	-112.776	46.881	0.248	-251.139	25.588	-110.609	45.631	0.236	-245.285	24.067
	Stage 4	-130.583*	43.644	0.044	-259.393	-1.774	-141.072*	42.631	0.015	-266.896	-15.248
Mesothelioma Stage 2	Unexposed (nad)[a]	5.756	55.475	1.000	-157.969	169.481	-51.278	55.059	1.000	-213.780	111.225
	Exposed (nad)[a]	-26.534	52.739	1.000	-182.187	129.119	-46.758	51.442	1.000	-198.585	105.069
	Stage 1	-8.917	62.336	1.000	-192.893	175.060	-16.127	60.648	1.000	-195.126	162.871
	Stage 3	-121.692	61.627	0.734	-303.576	60.191	-126.736	59.956	0.526	-303.692	50.219
	Stage 4	-139.500	59.202	0.283	-314.225	35.225	-157.200	57.745	0.101	-327.630	13.231
Mesothelioma Stage 3	Unexposed (nad)[a]	127.448*	37.276	0.010	17.435	237.462	75.459	37.636	0.684	-35.621	186.539
	Exposed (nad)[a]	95.159	33.068	0.063	-2.437	192.754	79.978	32.312	0.205	-15.389	175.346
	Stage 1	112.776	46.881	0.248	-25.588	251.139	110.609	45.631	0.236	-24.067	245.285
	Stage 2	121.692	61.627	0.734	-60.191	303.576	126.736	59.956	0.526	-50.219	303.692
	Stage 4	-17.808	42.625	1.000	-143.609	107.994	-30.463	41.563	1.000	-153.133	92.207
Mesothelioma Stage 4	Unexposed (nad)[a]	145.256*	33.112	0.000	47.530	242.982	105.922*	33.233	0.023	7.836	204.008
	Exposed (nad)[a]	112.966*	28.292	0.001	29.466	196.467	110.441*	27.660	0.001	28.804	192.079
	Stage 1	130.583*	43.644	0.044	1.774	259.393	141.072*	42.631	0.015	15.248	266.896
	Stage 2	139.500	59.202	0.283	-35.225	314.225	157.200	57.745	0.101	-13.231	327.630
	Stage 3	17.808	42.625	1.000	-107.994	143.609	30.463	41.563	1.000	-92.207	153.133

[a] no apparent disease
[b] These p-values are used in Table 5.

The estimated marginal means and the standard errors of the marginal means were calculated, and the Bonferroni method was used for pairwise comparison of the groups.
